# Silver Nanoforms as a Therapeutic Agent for Killing *Escherichia coli* and Certain ESKAPE Pathogens

**DOI:** 10.1007/s00284-016-1034-8

**Published:** 2016-04-16

**Authors:** A. Kedziora, K. Korzekwa, W. Strek, A. Pawlak, W. Doroszkiewicz, G. Bugla-Ploskonska

**Affiliations:** Department of Microbiology, Institute of Genetics and Microbiology, University of Wrocław, Przybyszewskiego 63-77, 51-148 Wrocław, Poland; Institute of Low Temperature and Structure Research, Polish Academy of Sciences, Okolna 2, Wrocław, Poland

## Abstract

The scope of this study included the preparation of silver nanoforms with high antimicrobial efficacy, low cost, and ease of application. The term ‘silver nanoforms’ refers to silver located on the amorphous or crystalline titanium dioxide (TiO_2_). Silver nanoforms may be used as an alternative to antibiotics in killing bacteria. Pure and silver-incorporated titanium (used as a carrier) was prepared using the sol–gel-modified method. Physical and chemical properties of the samples were described, and the antibacterial activity was indicated using the following strains of bacteria: *Staphylococcus aureus*, *Klebsiella pneumoniae* (ESKAPE pathogens), and *Escherichia coli*. The results have shown that the antibacterial activity of silver nanoforms with amorphous TiO_2_ is much better than that in the samples based on anatase (crystalline TiO_2_). The sensitivity of the tested bacteria to silver nanoforms depends on physical and chemical properties of the nanoforms and individual characteristics of the bacteria. For the first time, significant participation of amorphous TiO_2_ in antibacterial compounds has been described through this study.

## Introduction

Silver nanoforms are nanocompounds of silver located on the amorphous or crystalline titanium dioxide (TiO_2_). TiO_2_ is used as a carrier for biologically active silver (ions Ag^+^ and nanoparticles Ag^0^), causing (1) prevention of silver ions inactivation by environmental factors (such as NaCl), (2) prevention of silver nanoparticles aggregating with each other, (3) increase in the surface area (*S*_BET_) of silver nanoforms. The above points have one purpose: better (higher and faster) antibacterial activity of silver nanocompounds. TiO_2_ appears in the amorphous form and the following polymorphic crystalline ones: anatase, rutile, and brookite. Anatase is known for its highest photocatalytic properties, and for this reason, it has a potential for various biomedical and environmental applications [15, 20, 30]. Photocatalysis is a process during which, in a water environment, radicals are generated under the influence of UV light [4]. Radicals change the metabolism of bacteria cells, which causes their death. Up to now (October 2015), over 20,000 results in the PubMed have focused on the crystalline TiO_2_ (especially anatase) and its photocatalysis process. About 30 of them, concentrate on the comparison of their antibacterial efficacy, but only one refers to amorphous TiO_2_ as a carrier for silver and describes its potential antibacterial applications [7]. Research on bacterial resistance to antibacterial agents is an important trend in contemporary microbiology and the medical area. During the last six decades, resistance to antibiotics has increased, and when antibiotics were introduced in the 1950s, researchers expected that they would eradicate all bacterial diseases. In the early 1960s, resistance to penicillin among Gram-positive bacteria was noted, and the same phenomenon was soon observed in Gram-negative bacteria [24]. Multidrug resistance (MDR) in both Gram-positive and Gram-negative bacteria and extremely drug-resistant bacteria (XDR) is an important issue in the treatment of infectious diseases [22]. Antibiotic resistance control is a long-term international program which surveys MDR bacteria. Many international reports indicated that the resistance of bacteria to antibiotic drugs is still high [6]. *Klebsiella pneumoniae* as a nosocomial pathogen that also causes septicemia in newborns is increasingly multidrug-resistant [22]. MDR *Staphylococcus aureus* complicates the treatment of healthcare-associated infections. Also, carbapenems resistance in Gram-negative bacteria has been rapidly increasing [25]. Moreover, both *K. pneumoniae* and *S. aureus* belong to ESKAPE pathogens (together with *Enterococcus faecium*, *Acinetobacter baumannii*, *Pseudomonas aeruginosa*, and *Enterobacter* spp.). The ESKAPE group describes pathogens responsible for hospital infections, which are most difficult to treat. However, *E. coli* causing bacteremia infections and sepsis showed increasing resistance to fluoroquinolones [24]. The spread of resistance among pathogens is becoming a serious problem [14], and therefore searching for alternative drugs, such as those containing nanocomposites doped with silver, is very desirable. Silver ions and silver nanoparticles show a broad spectrum of antibacterial activity and indicate an oligodynamic effect [11, 18, 19]. Silver has been known for its antibacterial activity since ancient times, but the development of bionanotechnology has given us possibilities for using it as an active biomedical factor [16]. The toxicity of silver compounds depends on the bioavailability of silver, and silver ions rapidly bind to factors in the environment (such as Cl^−^) or aggregate with each other and eventually become unable to kill bacteria. The presence of a carrier (such as titanium dioxide) acts as a ligand for silver ions or nanoparticles and preservatives before decreasing the availability of silver [16]. Kedziora et al. [12] proposed the synthesis of TiO_2_/Ag compounds, where anatase indicated very good antibacterial efficacy. We modified the previous method, and in this work, other nanoforms of TiO_2_ were prepared—amorphous and crystalline (anatase) doped with silver—and their antibacterial activity was compared, with and without UV irradiation. This work proves excellent antibacterial activity of the nanoforms based on the amorphous TiO_2_—TiO_2_/Ag^+^ and TiO_2_/Ag^0^ (even without UV), much better than that based on the anatases—TiO_2a_/Ag^+^ and TiO_2a_/Ag^0^ (with UV irradiation). The amorphous nanoforms have a potential to be exploited in numerous biomedical, pharmaceutical, and industrial applications as an antimicrobial agent (e.g., for hard-to-heal wound infections as cream, ointment, bandages, disinfectants, etc., or as filters and preservatives in cosmetics). In order to maintain its excellent antibacterial activity, no additional factors such as UV irradiation are required.

## Materials and Methods

### Production of Pure Amorphous and Crystalline TiO_2_ and TiO_2_ Doped with Silver (TiO_2_/Ag^+^, TiO_2a_/Ag^+^, TiO_2_/Ag^0^ and TiO_2a_/Ag^0^) Nanocompounds

All used reagents were described in a study by Kedziora et al. [12]. Titanium *n*-butoxide [Ti(O-Bu)], hydrofluoric acid, and ammonium hydroxide were added as described by Kedziora et al. [12]. The duration of stirring was established experimentally. In the previous study [12], diamminesilver(I) [Ag(NH_3_)_2_]^+^ was added during the above procedure to form nanosilver grain. In this work, we (1) finished the gel synthesis (by washing with methyl alcohol and water, drying at 80 °C, and transforming to anatase, if necessary); and then (2) treated the powder of amorphous or crystalline TiO_2_ with diamminesilver(I) and a reducing factor (glucose) during impregnation and reduction. After step 2, we received nanocompounds of amorphous and crystalline: TiO_2_—TiO_2_/Ag^+^, TiO_2a_/Ag^+^ (as an impregnation effect) and TiO_2_/Ag^0^ and TiO_2a_/Ag^0^ (as a reduction effect). To prepare the anatase, amorphous TiO_2_ was calcinated under the same conditions as described previously [12]. The content of silver (Ag^+^ or Ag^0^) was measured by dissolving the composite in acetic acid or nitric acid accordingly, and then measured using atomic absorption spectroscopy (AAS, Perkin Elmer 1100). Results of nanoforms characteristics are presented in Table 1.

**Table 1:** Physicochemical description of prepared samples

Sample	Kind of TiO_2_	Size of TiO_2_ (nm)	Size of silver nanoparticles (nm)	Content of silver (wt%)
TiO_2_	Amorphous	≤100	n/a	n/a
TiO_2_/Ag^0^	Amorphous	≤100	20	10
TiO_2_/Ag^+^	Amorphous	≤100	n/a	10
TiO_2a_	Anatase	≤40 (aggregate)	n/a	n/a
TiO_2a_/Ag^0^	Anatase	≤40 (aggregate)	≤10	1
TiO_2a_/Ag^+^	Anatase	≤40 (aggregate)	n/a	1

*n/a* not applicable

### Characteristics of Pure TiO_2_ and TiO_2_ Doped with Silver (TiO_2_/Ag^+^, TiO_2a_/Ag^+^, TiO_2_/Ag^0^ and TiO_2a_/Ag^0^) Nanocomposites

The description of morphology is one of the basic features of materials in the nanoscale. The samples were characterized as follows: size and shape—with transmission electron microscopy (TEM, Philips CM20 Super Twin); crystalline structure—with X-ray diffraction (XRD, Stöe); porosity—with nitrogen adsorptions isotherms (Autosorb 1-C, Quantachrome Instruments); and content of silver—with atomic absorption spectroscopy (AAS, Perkin-Elmer). Diffraction patterns (degrees and intensity of peaks measured with XRD) were compared with the referenced patterns from the database of diffraction patterns: International Centre for Diffraction Data (PDF2 Database ICDD). Porosity measured with N_2_ adsorption and desorption provides information about the pore size and the corresponding surface area. Porosity size is of fundamental importance in developing the surface area—a basic feature in nanotechnology. The smaller the pore sizes observed the larger the surface area described. A shape of the isotherm confirms a pore size. To measure the silver content in the nanocompounds with AAS, the solubility of silver ions in citric acid and that of silver nanoparticles in the nitric acid were used. The results obtained with the above techniques make the interpretation of antibacterial activity of silver nanomaterials easier. The photocatalytic activity of nanocompounds was measured using a model reaction of estimated decomposition of methyl blue (at room temperature, RT) [28].

### Bacteria Strains

In all, 10 bacteria strains were used for antimicrobial study—three reference strains from American Type Culture Collection: *Staphylococcus aureus* ATCC 6538 (Gram-positive bacteria), *Escherichia coli* ATCC 11229 (Gram-negative bacteria), and *Klebsiella pneumoniae* ATCC 4352 (Gram-negative bacteria); and seven of the same genera strains, isolated from infected wounds purchased from the DiaLab Medical Laboratory. The antibiogram is presented in Table 2. Clinical isolates of *E. coli* 475, 555, 574, and *K. pneumoniae* 626 strains are resistant to penicillin, penicillin with inhibitor, and cefalosporins, the second generation drugs frequently used as the first applied antibiotics. *S. aureus* strains (173, 187) are methicillin resistant (MRSA), resistant to chinolon, trimethoprim–sulfamethoxazole, macrolides, and lincosamides. *S. aureus* 298 is a methicillin-sensitive (MSSA) strain. The antibiogram was done according to Kirby-Bauer disk diffusion-susceptibility test protocol [5].

**Table 2:** Antibiogram of bacterial isolates

Bacterial isolates	Am	Amc	Cxm	Ctx	Caz	Atm	An	Cip	SXT	Imp	Mem	TZP			
*E. coli* 475^a^	S	R	R	S	S	S	S	S	S	S	S	S			
*E. coli* 555^a^	S	R	R	S	S	S	S	S	S	S	S	S			
*E. coli* 574^a^	S	R	R	S	S	S	S	S	S	S	S	S			
*K. pneumoniae* 626^a^	S	R	R	S	S	S	S	S	S	S	S	S			

	P	Amc	Cxm	Ctx	Caz	Ge	An	Cip	Sxt	Imp	Mem	E	Cc	Va	Fox
*S. aureus* 173^a^	R	R	R	R	R	S	S	R	R	R	R	R	R	S	R
*S. auerus* 298^a^	R	S	S	S	S	S	S	S	R	S	S	S	S	S	S
*S. auerus* 187^a^	R	R	R	R	R	R	R	R	R	R	R	R	R	S	R

*S* sensitive, *R* resistant, *Am* ampicillin, *AMC* amoxicillin-clavulanic acid, *CXM* cefuroxime, *CTX* cefotaxime, *CAz* ceftazidime, *AN* amikacin, *Ge* gentamicin, *ATM* aztreonamum, *CIP* ciprofloxacin, *Imp* imipenem, *Mem* meropenem, *Tzp* piperacillin–tazobactam, *SXT* trimethoprim–sulfamethoxazole, *P* penicillin, *E* erytromycin, *CC* clindamycin, *VA* vancomycin, *FOX* cefoxitin

^a^Bacterial isolates, numbered by Medical Laboratory DiaLab

### Antibacterial Effect of Nanocomposites: TiO_2_/Ag^+^, TiO_2a_/Ag^+^, TiO_2_/Ag^0^ and TiO_2a_/Ag^0^

The antibacterial effect of amorphous (TiO_2_/Ag^+^, TiO_2_/Ag^0^) and crystalline (TiO_2a_/Ag^+^, TiO_2a_/Ag^0^) nanocompound was determined by minimal inhibitory concentration (MIC) values according to the reference methods of Clinical and Laboratory Standards Institute (CLSI) for the determination of MICs of aerobic bacteria by broth microdilution [8, 27] with minor modification. The stock of the antimicrobial agent’s dilution was prepared in microplates, and the final inoculum was 10^6^ CFU/ml per spot. Nanoforms-free spots with a medium, pure TiO_2_ and TiO_2a_ and cultures were used as growth controls. To study the bacterial inactivation effect of titanium nanocomposites after UV irradiation, the tested samples were irradiated with UV light (Philips, 30 W/cm^2^) for 30 min.

## Results and Discussion

### Synthesis and Characterization of Silvered TiO_2_

**Fig. 1:**
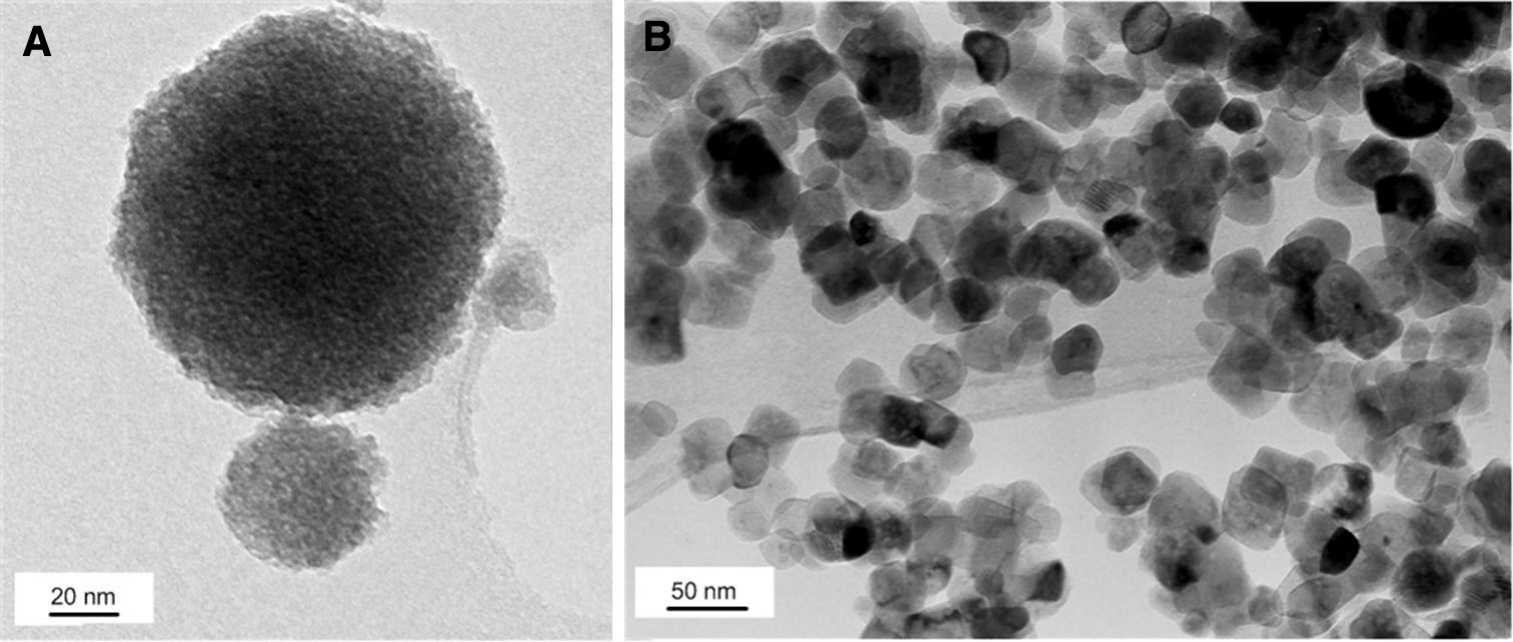
TEM images of amorphous TiO_2_ (**a**) and crystalline TiO_2_ (**b**)

Sol–gel synthesis is one of the most popular methods used for nanocompounds production [2, 3, 23] and its modification leads to obtaining various products with differences in physicochemical and biological properties. In comparison with the study by Kedziora et al. [12], we showed that a source of silver [diamminesilver(I)] may be added during the synthesis of powder forming silver grain and to the ready gel (this work). The way of synthesizing has an impact on the surface area and antibacterial efficacy. In Fig. 1, the TEM images of the prepared basic samples are shown. Figure 1a presents amorphous TiO_2_, and anatase (TiO_2a_) is presented in Fig. 1b. The size of amorphous TiO_2_ is less than or equal to 100 nm. Calcination of amorphous TiO_2_ caused a decrease in grain size (from 100 to 40 nm), but silver grain embodied to TiO_2_ structure [12] decreased the surface area (*S*_BET_). The specific surface area of the powder decreased from 424.0 ± 8.0 m^2^/g (for amorphous TiO_2_) to 52.8 ± 0.2 m^2^/g (for crystalline TiO_2a_) (Table 3). High reduction of surface area is probably related to the aggregation of TiO_2_ during annealing.

**Table 3:** Surface area (*S*_BET_) and pore size get with nitrogen adsorptions

Sample	*S*_BET_ (m^2^/g)	Pore size (nm)
TiO_2_	424.0 ± 8.0	4
TiO_2a_	52.8 ± 0.2	22

**Fig. 2:**
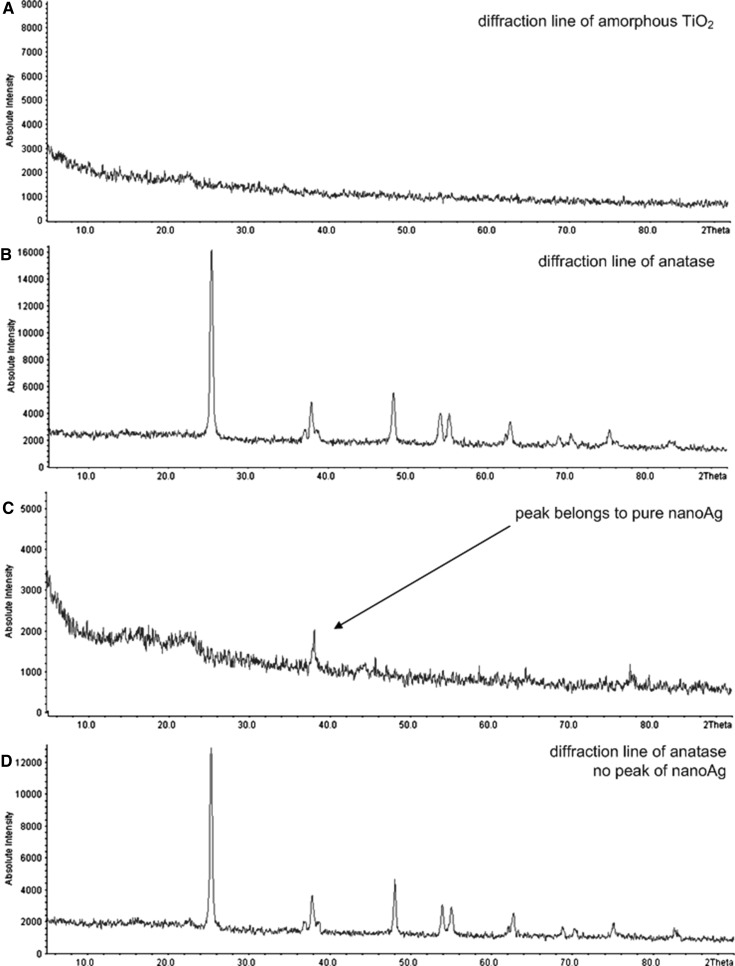
XRD patterns of: **a** amorphous TiO_2_, **b** crystalline TiO_2a_, **c** amorphous TiO_2_/Ag^0^ and **d** crystalline TiO_2a_/Ag^0^

**Fig. 3:**
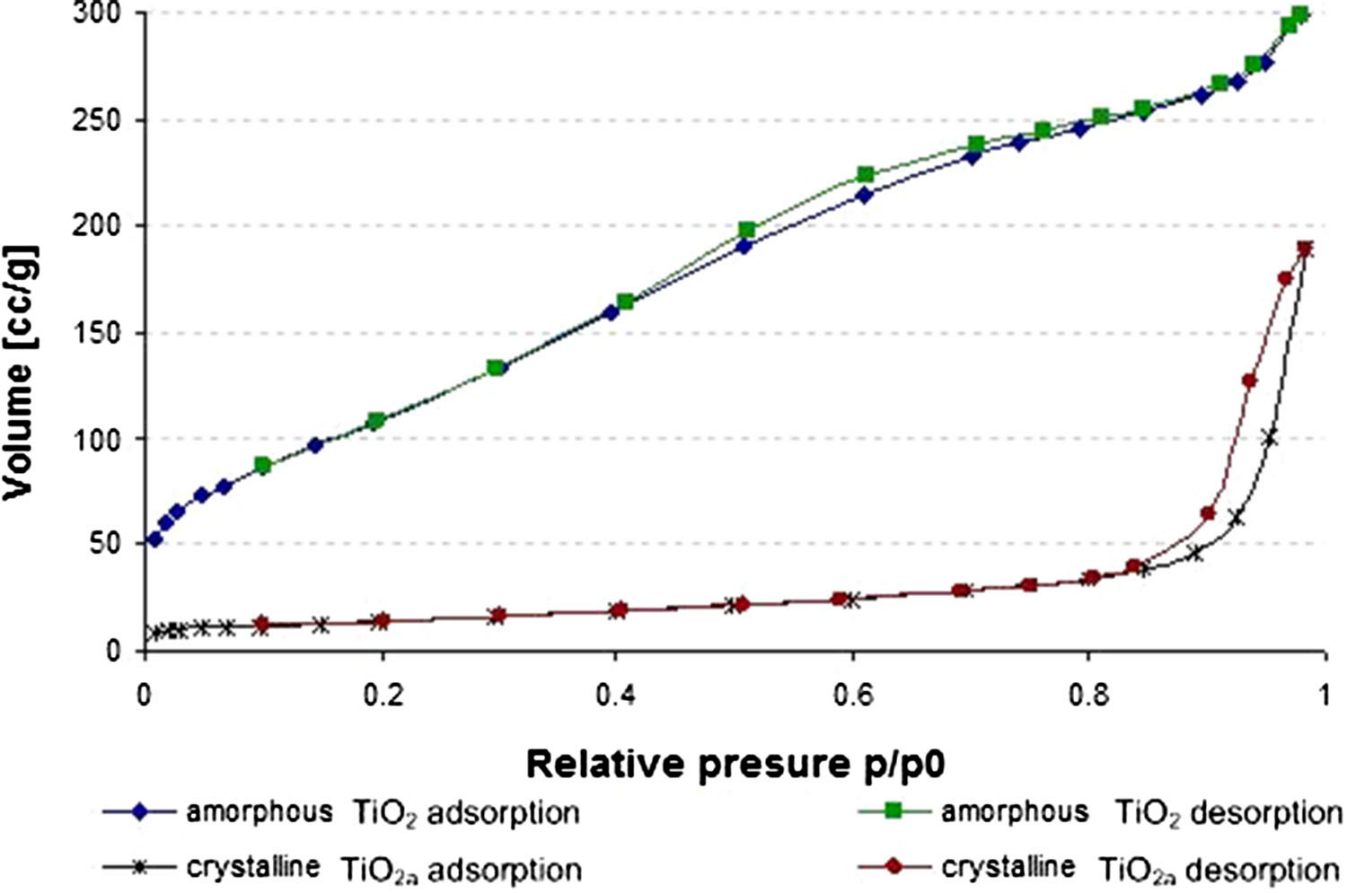
N_2_ adsorption and desorption by amorphous TiO_2_ and crystalline TiO_2a_

In a study by Kedziora et al. [12], adding diamminesilver(I) to the sol of TiO_2_ was proposed. It caused a decrease in the size of silver nanoparticles to less than 5 nm [12]. Calcination of amorphous titanium (TiO_2_) did not change the silver crystallite size. X-ray diffraction lines of amorphous and crystalline TiO_2_ are shown in Fig. 2. All of these are own results, and the obtained data were compared with the ICDD database. We confirmed that amorphous TiO_2_ powder calcinated at 400 °C is already well crystallized to anatase (Fig. 2b, d). All reflections in the diffraction patterns shown in Fig. 2b were identified as belonging to anatase (according to ICDD Database No. 99-200-3744, 38.78 2Theta). In the pattern observed in Fig. 2a, c, the amorphous nature of TiO_2_ was also proved [lack of clear peaks and shoulder peak around 23° (2*θ*)]. In Fig. 2c, there is a visible peak showing the presence of pure nano Ag [peak around 38° (2*θ*), with silver content estimated at 10 wt% with AAS, Table 1] embedded in amorphous titanium samples (according to ICDD Database No. 99-101-3086, 38.25 2Theta). In Fig. 2d (diffraction lines of TiO_2a_/Ag^0^), peaks of anatase can be observed. However, no visible silver peak in Fig. 2d probably results from (1) a low content of silver (estimated at 1 wt% for TiO_2a_/Ag^0^, see Table 1, proved with AAS) or (2) interference of the anatase and nanosilver peaks. N_2_ adsorption–desorption isotherms of the amorphous and crystalline TiO_2_ are illustrated in Fig. 3. The isotherms of adsorption and desorption do not cover each other, which confirms that the prepared materials (amorphous TiO_2_ and crystalline TiO_2a_) are porous. From the shape of hysteresis, it can be concluded that the material is mesoporous (the pore diameter of titanium dioxide is between 2 and 50 nm) [21]. Photocatalytic properties of the prepared anatase were experimentally confirmed by measuring the decomposition of methyl blue. Average time of decomposition of methyl blue by crystalline anatase (TiO_2a_) was estimated at 90 min.

The knowledge of the nanocarrier morphology (size, shape, porosity, surface area, and crystal structures) is a fundamental issue and has an important impact on the following aspects: (1) silver content in the nanocompounds, (2) antibacterial efficacy of silver nanocompounds, (3) interaction of bacteria cells with silver nanocompounds, (4) antibacterial mechanism of action, and (5) mechanism of potential bacterial resistance to silver nanomaterials. Furthermore, this knowledge is fundamental for further research concerning the influence of physicochemical properties of the compounds on the answer of bacteria cells.

### Antibacterial Activity

Results of the antibacterial efficacy of silver nanocomposites are summarized in Tables 4 and 5. We checked antimicrobial activity of temporary forms—TiO_2_ impregnated with silver ions Ag^+^ (TiO_2_/Ag^+^ and TiO_2a_/Ag^+^) and permanent forms—TiO_2_ doped with silver nanoparticles Ag^0^ (TiO_2_/Ag^0^ and TiO_2a_/Ag^0^) against Gram-positive and Gram-negative bacteria. Pure TiO_2_ and TiO_2a_ did not show antibacterial activity in the applied concentration (1024 µg/ml). The best efficacy was indicated in the sample based on amorphous titanium dioxide used as a carrier for silver ions (TiO_2_/Ag^+^) where MIC was equal for ions with and without UV irradiation and reached 0.05 µg/ml. Excellent efficacy is indicated in the silver nanoparticles (Ag^0^) also located on the amorphous TiO_2_ (TiO_2_/Ag^0^). Also, MIC of TiO_2_/Ag^0^ comes to 0.4 µg/ml (the same values for UV irradiation and without it). Bionanotechnologists seem to overlook the amorphous TiO_2_, but we proved its excellent properties as a carrier for antibacterial agents such as silver (both silver ions and nanoparticles). It is very attractive for medical and biological applications—shows a very good antibacterial efficacy even without UV treatment. Annealing to the anatase structure may be limited and stopped after the amorphous TiO_2_ phase. Silver nanoforms based on the crystalline TiO_2a_ indicated lower antibacterial activity than those based on an amorphous one even after UV treatment. MIC values for TiO_2a_/Ag^+^ reached the level of 0.8–3.2 µg/ml without UV irradiation and 0.4–1.6 µg/ml for TiO_2a_/Ag^0^ after UV treatment. However, for TiO_2a_/Ag^0^ MIC values were established as 25.6–51.2 µg/ml without UV and 12.8–25.6 µg/ml after UV irradiation. It is worth underlining that the tested clinical isolates of *E. coli* (475, 555, 574) and *K. pneumoniae* 626 are resistant to penicillin and cefalosporins, and two of the tested *S. aureus* are MRSA bacteria strains (*S. aureus* 173, and 187, Table 2). When compared with the bacterial sensitivity and resistance to antibiotics, the obtained MIC values for silver nanoforms result from a different antibacterial mechanism of action. Yanez et al. [29] confirmed a good reduction of bacteria by TiO_2_ nanotubes doped with polyethylene composites to over 40–99 % after UV light and about 28–43 % after white light irradiation. Results of our study confirm that the major reason for high antibacterial activity of silver nanoforms is the surface area, higher in amorphous than crystalline titanium dioxide. The increased *S*_BET_ of anatase is due to lower bacterial susceptibility. Li et al. [15] also showed that a diameter of TiO_2_ is one of the most important factors in the antibacterial efficacy. In contrast to our study, Li et al. [15] indicated that anatase has higher antibacterial activity than amorphous titania. In studies by Jasiorski et al. [10] and Wiglusz et al. [26], very good efficacy of silver nanoparticles deposited on other amorphous compounds such as silica surface [10] and hydroxyapatites [26] was proved. Amorphous TiO_2_ has a potential to become a perfect carrier for antibacterial metals such as silver. In comparison with the previous research [12] the MIC values for the TiO_2_ and TiO_2a_ impregnated with silver ions (TiO_2_/Ag^+^, TiO_2a_/Ag^+^) on *S. aureus*, *E. coli*, and *K. pneumoniae* were significantly lower than the MIC values obtained for Ag nanoparticles (Tables 4, 5), so antibacterial sensitivity depends on both the kinds of carrier and silver oxidation. Silver ions react quickly but, unlike silver nanoparticles, are rapidly inactivated by environment factors, e.g., Cl^−^. Gao et al. [7] also proposed the production of amorphous TiAg_2_O which revealed very effective antibacterial properties, cytocompatibility, and long lasting effectivity. Moreover, Zhao et al. [31] proved the long-term antibacterial ability of silver nanoparticles and biointegration. In contrast to Keleher et al. [13], we demonstrated less sensibility of Gram-negative bacteria to silver nanoparticles immobilized on the anatase (TiO_2a_/Ag^0^) than in the case of Gram-positive strains. However, Jamuna-Thevi et al. [9] and Mirzajani et al. [17] proved good activity of silver nanoparticles embedded on the anatase against *S. aureus*. They also proved that the minimum antibacterial inhibitory concentration of Ag^0^ was less than the maximum cytotoxic concentration. Abdal-hay et al. [1] suggested that coating the Ti with different polymer layers causes more efficacy against bacteria.

**Table 4:** MIC (µg/ml) of amorphous TiO_2_ doped with silver (TiO_2_/Ag^+^ and TiO_2_/Ag^0^) with and without UV irradiation

Bacteria strains	MIC [µg/ml] TiO_2_/Ag^+^		MIC [µg/ml] TiO_2_/Ag^0^	
	Without UV	With UV	Without UV	WITH UV
*S. aureus* ATCC 6538	0.05	0.05	0.4	0.4
*S. aureus* 173^a^	0.05	0.05	0.4	0.4
*S. aureus* 187^a^	0.05	0.05	0.4	0.4
*S. aureus* 298^a^	0.05	0.05	0.4	0.4
*E. coli* ATCC 11229	0.05	0.05	0.4	0.4
*E. coli* 475^a^	0.05	0.05	0.4	0.4
*E. coli* 555^a^	0.05	0.05	0.4	0.4
*E. coli* 574^a^	0.05	0.05	0.4	0.4
*K. pneumoniae* ATCC 4352	0.05	0.05	0.4	0.4
*K. pneumoniae* 626^a^	0.05	0.05	0.4	0.4

^a^Bacterial isolates

**Table 5:** MIC (µg/ml) of anatase doped with silver (TiO_2a_/Ag^+^ and TiO_2a_/Ag^0^) with and without UV irradiation

Bacteria strains	MIC (µg/ml) TiO_2a_/Ag^+^		MIC (µg/ml) TiO_2a_/Ag^0^	
	Without UV	With UV	Without UV	With UV
*S. aureus* ATCC 6538	0.8	0.4	25.6	12.8
*S. aureus* 173^a^	1.6	0.8	25.6	12.8
*S. aureus* 187^a^	1.6	0.8	25.6	12.8
*S. aureus* 298^a^	1.6	0.8	25.6	12.8
*E. coli* ATCC 11229	3.2	1.6	25.6	12.8
*E. coli* 475^a^	3.2	1.6	51.2	25.6
*E. coli* 555^a^	1.6	0.8	51.2	25.6
*E. coli* 574^a^	3.2	1.6	25.6	12.8
*K. pneumoniae* ATCC 4352	3.2	1.6	51.2	25.6
*K. pneumoniae* 626^a^	3.2	1.6	51.2	25.6

^a^Bacterial isolates

In summary, we propose that silver nanoforms based on the amorphous TiO_2_ have a potential to be used in the inhibition of growth or treatment of an infection caused by MDR *E. coli* and *K. pneumoniae* strains or MRSA *S. aureus*.

## Conclusions

Various modifications of the sol–gel technology make it possible to synthesize different silver nanoforms with great possibilities to be used in the therapy of infections. Titanium dioxide with its physical and chemical properties may be used as a carrier of silver ions and nanoparticles. Amorphous TiO_2_ is an ideal carrier for biologically active silver, both ions and nanoparticles, even without UV irradiation. The surface area of the carrier has an impact on the antibacterial efficacy. We have proven an alternative way to antibiotics for killing bacteria and have proposed amorphous TiO_2_ as a carrier for biologically and medically active silver.

## Abbreviations

[Ag(NH_3_)_2_]^+^: Diamminesilver(I)
AAS: Atomic absorption spectroscopy
ATCC: American Type Culture Collection
CFU/ml: Colony-forming units per ml
CLSI: Clinical and Laboratory Standard Institute
ESKAPE: Group of six pathogens (*Enterococcus faecium*, *Staphylococcus aureus*, *Klebsiella pneumoniae*, *Acinetobacter baumannii*, *Pseudomonas aeruginosa*, and *Enterobacter* spp.)
ICDD: International Centre for Diffraction Data (PDF2 Database ICDD)
MDR: Multidrug resistance
MIC: Minimal inhibitory concentration
MRSA: Methicillin-resistant *S. aureus*
MSSA: Methicillin-sensitive *S. aureus*
RT: Room temperature
*S*_BET_: Surface area
TEM: Transmission electron microscopy
Ti(O-Bu): Titanium *n*-butoxide
TiO_2_: Amorphous titanium dioxide
TiO_2_/Ag^+^: Amorphous titanium dioxide doped with silver ions
TiO_2_/Ag^0^: Amorphous titanium dioxide with silver nanoparticles
TiO_2a_: Crystalline titanium dioxide (anatase)
TiO_2a_/Ag^+^: Crystalline titanium dioxide (anatase) doped with silver ions
TiO_2a_/Ag^0^: Crystalline titanium dioxide (anatase) with silver nanoparticles
UV: Ultraviolet light
XDR: Extremely drug resistance
XRD: X-ray diffraction
